# Effectiveness of Swimming Program in Adolescents with Down Syndrome

**DOI:** 10.3390/ijerph18147441

**Published:** 2021-07-12

**Authors:** Alicja Naczk, Ewa Gajewska, Mariusz Naczk

**Affiliations:** 1 Department of Physical Education and Sport, Faculty of Physical Culture in Gorzow Wielkopolski, University School of Physical Education in Poznan, 66-400 Gorzow Wielkopolski, Poland; a.naczk@awf-gorzow.edu.pl; 2 Department of Developmental Neurology, Poznan University of Medical Sciences, 60-355 Poznan, Poland; ewagajewska@ump.edu.pl; 3 Institute of Health Sciences, Collegium Medicum, University of Zielona Gora, 65-417 Zielona Gora, Poland

**Keywords:** swimming program, aerobic capacity, muscle strength, body composition, Down syndrome

## Abstract

The aim of this study was to estimate the influence of a 33-week swimming program on aerobic capacity, muscle strength, balance, flexibility, and body composition of adolescents with Down syndrome (DS). Twenty-two adolescents diagnosed with DS were randomly allocated into the training group (T) and the control group (C). The T group participated in 33 weeks of water-based exercise and a swimming program while the control group maintained their normal daily activity. Following thirty-three weeks of swimming program, body mass, body fat, and BMI of the T group decreased significantly (from 56.8 ± 7.97 kg to 55.0 ± 7.11 kg, from 15.1 ± 4.47 kg to 13.2 ± 3.92 kg, and from 25.1 ± 2.37 to 24.0 ± 2.05, respectively) while a significant increase was recorded in C (from 57.3 ± 8.43 kg to 59.7 ± 8.29 kg, from 14.5 ± 2.76 kg to 16.0 ± 3.11 kg, and from 25.4 ± 2.46 to 26.0 ± 2.72, respectively). Moreover, significant improvement in aerobic capacity in the T group was noted; VO_2_max (mL/kg/min) increased by 16.3% in T and decreased by 4.8% in C. Improvement in static arm strength, trunk strength and endurance/functional strength were noted in T, while the parameters did not change in C. The speed of arm movement, balance and flexibility did not change following the intervention. Also, the aquatic skills improved significantly in the training group. Changes in C were not significant. The results of our study indicate that 33-week swimming program significantly improved health status and swimming skills in adolescents with DS.

## 1. Introduction

Adolescents with Down syndrome (DS), compared to their healthy peers, are more likely to suffer from overweight and obesity [1]. Overweight/obesity and low physical activity significantly increase the risk of mortality and many serious diseases [2]. Cardiovascular diseases (mitral value prolapse, endocarditis, atherosclerosis and congestive heart failure [3]), pulmonary hypoplasia, muscle hypotonia, osteoporosis, arthritis, osteoarthritis, and diabetes mellitus [4,5] are more common in people with DS compared to healthy ones. Moreover, DS results in low intelligence quotient and poor self-care skills [6]. Adolescents with DS have lower aerobic capacity than adolescents without DS. Moreover, VO_2_max and HRmax achieved by adolescents with DS is also lower than in adolescents with intellectual disability yet without DS [7]. It is important to point out that low aerobic capacity has also been strongly linked to morbidity and mortality among individuals with DS [6,8]. Patients with Down syndrome have poor muscle strength, agility, and balance compared to similarly age-matched peers [9,10]. It is recommended that to maintain/improve health status, young people should engage in physical activity of moderate-to-vigorous intensity for an average of at least 60 min per day throughout the week. This can include all forms of activity such as physical education, active travel, after-school activities, play, and sports [11]. However, it is estimated that 80% of teenagers worldwide are not sufficiently active [12]. Unfortunately, physical activity of people with DS when compared to that of their peers’ is usually lower [13]. Given the above, a higher than usual level of physical activity in people with DS is required. The influence of physical exercise/training on health status and functional capacity of DS patients were tested. However, the results of the studies were not consistent. Some authors stated that training young people with DS led to an improvement in cardiac efficiency, increased maximum ventilation, cardiorespiratory, muscle strength, and aerobic work capacity improvement [14,15]. Ilinca et al. [16] stated that the six-month program based on exercise therapy and aquatic therapy appears to have had a positive impact on the physical health of people with DS, reducing the potential health risks associated with low fitness and a sedentary lifestyle. On the other hand, Millar et al. [17] and Pérez et al. [18] found no evidence to support the hypothesis that exercise might be useful intervention to improve aerobic capacity, health-related physical fitness, and quality of life in subjects with DS. Also, Suarez-Villadat et al. [19] concluded that physical activity of DS participants did not reflect improvement in physical fitness. Different models of training were tested. One of the methods was water-based physical activity. Water environment is usually well tolerated by youth and they participate in such exercises willingly. To our knowledge, there are not enough studies concerning the influence of a swimming program on the functional capacity, aerobic capacity, muscle strength, balance, and body composition of adolescents with DS. Ilinca et al. [16], Pérez et al. [18], Suarez-Villadat et al. [19] tested influence of different programs realized in water conditions on fitness and health status in people with DS. However, the results of their studies are not consisted. Therefore, further research on the usefulness of aquatic exercise for the health status of people with DS are needed.

The aim of this study was therefore to estimate the influence of a 33-week swimming program on aerobic capacity, muscle strength, balance, flexibility, and body composition of adolescents with DS. The secondary objective of the program was to improve their independence in the water medium and learning/improving their swimming ability.

## 2. Materials and Methods

### 2.1. Participants

**Figure 1:**
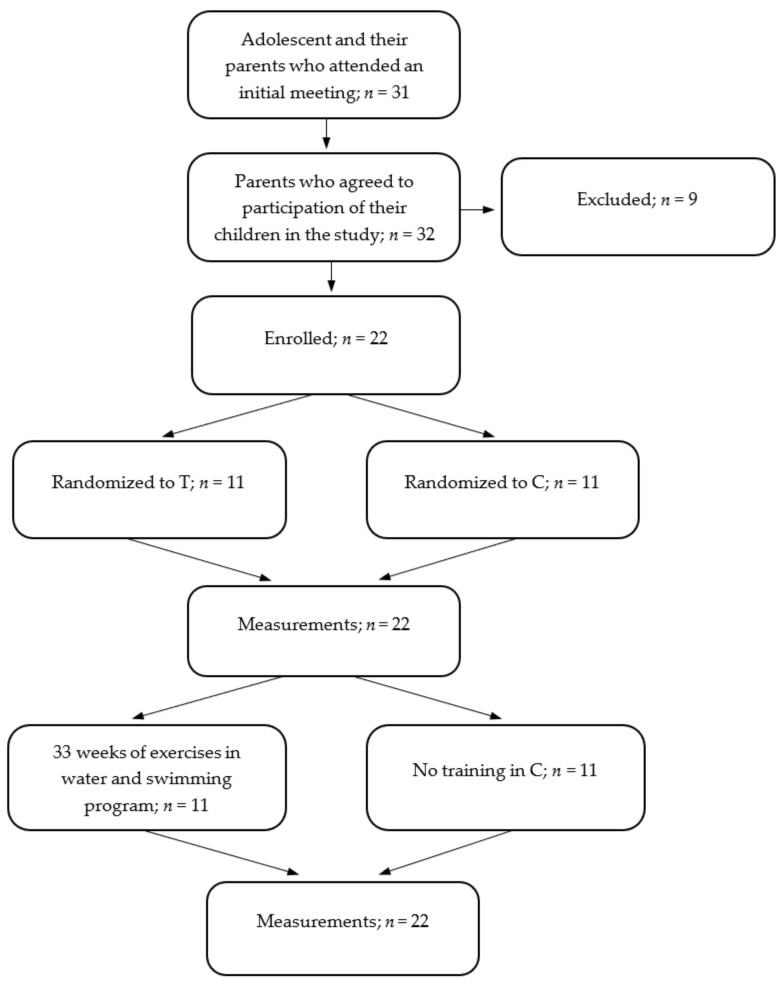
Flow diagram for study participants.

A group of 31 adolescents with Down syndrome (DS) and their parents attended the initial meeting. Still, nine subjects were excluded after the following criteria were applied: insufficient contact with the participants-they were unable to follow simple instructions, serious physical disability that might have interfered with the exercise program (serious heart disease, epilepsy, injury, hearing impairment). The study finally included 22 adolescents diagnosed with DS by a certified physician (14 boys and 8 girls). The participants were randomly allocated into two groups: the training group (T; *n* = 11; age, 14.9 ± 2.35 years; body mass, 56.8 ± 7.97 kg; height, 152 ± 8.65 cm) and the control group (C; *n* = 11; age, 14.4 ± 1.97 years; body mass, 57.2 ± 8.43 kg; height, 150 ± 12.0 cm) using the chit method. Each group included seven boys and four girls. The T group participated in 33 weeks of water-based exercise and a swimming program while the control group maintained their normal daily activity. The details are presented in the participants’ flow chart (Figure 1). All parents or legal guardians gave written, informed consent for their children to take part in the study. Moreover, the parents or legal guardians provided written, informed consent for their children images to be published. All procedures were approved by the Ethics Committee of Collegium Medicum, University of Zielona Gora, Poland (KB-UZ/5/2021, 31 March 2021), with the approval based on the Declaration of Helsinki.

### 2.2. Testing

To evaluate the influence of 33 weeks of exercise on body composition, bioelectrical impedance device (Tanita MC-980 MA, Tanita Corporation, Tokyo, Japan) was used. The participants (by means of a formal request communicated to their parents) were asked to maintain a normal state of hydration prior to measurement, and they were not allowed to exercise, or eat for 12 h preceding the measurements. The measurements were made in the morning, according to manufacturer’s guidelines.

### 2.3. Eurofit Test

Physical fitness was assessed using the Eurofit Test Battery. The following trials were performed:

- plate tapping-tests which measure the speed of limb movement
- handgrip test which measures static arm strength (using Takei 5401 dynamometer, Takei, Niigata, Japan)
- flamingo balance test which is a single leg balance test
- sit-and-reach flexibility test
- sit-ups in 30 s which measure trunk strength
- bent arm hang which measures muscular endurance/functional strength

The test battery was proven as a reliable tool to assess physical fitness in people with intellectual disabilities, with intraclass correlation coefficients between 0.94 and 0.99 [20].

### 2.4. Aerobic Capacity

Moreover, aerobic capacity (peak oxygen uptake-VO_2_ peak) was assessed using the maximum exercise test with the protocol validated for people with DS [21,22]. The test was performed on a treadmill (HP Cosmos, HP Cosmos Sports & Medical GMBH, Nussdorf-Traunstein, Germany). During the test, breath-by-breath oxygen uptake was continuously recorded using the Start 2000 ergospirometric system (MES, Krakow, Poland). Heart rate was continuously recorded during the test using the portable Polar Sport Tester heart rate telemetry device (Finland). Initially, the participants were walking at a comfortable pace for 4 min (4 km/h as a warm-up). This was followed by grade increments of 2.5% every 2 min until a 12.5% grade was achieved, after which only the speed was increased by 0.8 km/h every minute until exhaustion. The data acquired during the test were filtered every 10 s for analysis. Peak VO_2_ was chosen as the highest value (a 30-s average) observed during the last exercise stage. The test was completed if a participant was unable to maintain the treadmill speed, having a respiratory exchange ratio > 1.0, and a plateau in HR and/or oxygen uptake (VO_2_) with an increase in work rate.

### 2.5. Water Orientation Test Alyn 2 (WOTA2)

The aim of this evaluation is to assess the participant’s level of adjustment and function in water. Both the swimmer and the instructor were in the water at the time of testing. In addition to verbal instruction, the instructor demonstrated the task to be performed. Each item was attempted up to three times. A four-point ordinal scale (0–3 points) was developed for each skill based on the level of performance and functional independence [23]. When there was uncertainty as to which score to assign, the lower of the two possible scores was chosen. The scale was demonstrated to be reliable and valid [24]. The following measures were calculated: mental adaptation (max = 39 points), skills balance control movement (max = 42 points), and total score (max = 81 points).

### 2.6. Training Program

During the intervention period both groups continued with their normal everyday activities. The daily routine did not include systematic physical activity. Swimming training was performed by T group three times a week (on Monday and Friday between 4:30 p.m. and 6:00 p.m. and on Friday between 5:00 p.m. and 6:30 p.m.) for 33 weeks. At the start of the swimming program the level of swimming abilities was low, only four adolescents from T group could swim using the elementary technique (backstroke only). Attendance at all sessions was strictly monitored. The training program was divided into four stages:

- At the first stage, which lasted 4 weeks, the adolescents were getting used to the aquatic environment, and mainly water games were used to that effect. During this stage the participants learned how to submerge their heads under water, open their eyes under water, and lie in the water on their chests and backs. After this stage, the adolescents were visibly happy when they were entering the water, they showed no fear of the aquatic environment. At this stage swimming session lasted 70 min.
- At the second stage, which also lasted 4 weeks, water games were still carried out and the participants were taught to exhale into the water, slide on the chest and back, and make simple jumps into the water, at the end of this stage the participants had the ability to float on the water. At this stage swimming session lasted 80 min.
- At the third stage, lasting 15 weeks, the adolescents learned to swim in the four swimming styles: backstroke, crawl, breaststroke and butterfly, water games constituted 30% of the main part of the training session, learning to swim constituted 70% of the main part of the training session. At this stage swimming session lasted 90 min.
- The last 10-week stage was aimed at improving swimming skills. The exercises performed with and without the swimming equipment were focused on improving the swimming technique. During these training sessions, the adolescents usually swam 700 to 1000 m. At this stage swimming session lasted 90 min.

### 2.7. Training Session

Up to 11 adolescents participated in each training session. The training was conducted by two swimming coaches (one was in the water with the participants, the other one was involved in the training from the edge of the pool). In addition, there were also two volunteers in the water assisting the coaches. The structure of each training session was as follows:

- the warm-up outside the pool—10 min; and warm-up in water—10 min;
- the main part of the training session for the first 4 weeks lasted 30 min, the next 4 weeks—40 min; and in the following weeks—50 min;
- the recovery part (games in the water) lasted 20 min.

The condition for participation in the project was a minimum attendance of 90%. The training was performed in a swimming pool with a length of 25 m and depth from 1.0 m (first stage of training) to 1.8 m.

### 2.8. Statistical Analysis

The Shapiro-Wilk test was used to confirm the normal distribution of the data. Descriptive statistics, including means and standard deviations, were calculated. Paired *t*-tests were used to test for significant changes within groups, comparing values before and after training. The simple effect of training was defined as a relative increase in an analyzed variable after training compared with the before-training value, using the formula

(1) $$RI\;\left[\%\right]=\frac{x_{post}-x_{pre}}{x_{pre}}\;\times \;100$$

where *RI* stands for the relative increase and *x* is the measured value before (pre) and after (post) training.

The differences in relative increases between groups were tested with one-way ANOVA. If differences were detected, the Scheffé post hoc procedure was used to determine where the differences occurred. The level of significance was set at *p* ≤ 0.05. The effect size (ES) of the training was calculated using the independent two-sample *t*-test, and Cohen’s *d* was established. The scale presented by Cohen [25] indicates that d < 0.41 represents a small ES, 0.41–0.70 a moderate ES, and higher than 0.70 a large ES.

## 3. Results

None of the analyzed parameters differed significantly between the tested groups at the beginning of the experiment.

The exercise group exhibited significant decreases in body mass, body fat, and BMI; while the control group showed significant increases in these variables (Table 1). Moreover, relative changes in body mass, body fat, and BMI noted in T were significantly different than those observed in C (Table 2). Height increased significantly in both groups (Table 1). The training group showed a statistically significant increase in aerobic capacity, while the control group showed significant decreases in VO_2_max (Table 1). The effect sizes expressed by Cohen’s d values for VO_2_max, body mass, body fat, and BMI were high (Table 2). Following 33 weeks of training static arm strength, trunk strength and endurance/functional strength improved significantly in T (Table 3). However, the speed of limb movement, balance, and flexibility did not change following the intervention. There were no significant changes in the C group.

Before swimming program, the level of the swimming abilities in T and C were low and similar in both groups. The results of WOTA2 showed that both mental adaptation and skills balance control movement improved significantly in T group (from 8.45 ± 3.33 to 34.1 ± 5.58 and from 4.55 ± 7.79 to 31.3 ± 7.81, respectively) and remained unchanged in C (from 8.27 ± 2.05 to 8.73 ± 1.01 and from 7.45 ± 7.27 to 7.64 ± 6.05, respectively). The effect size of the training noted in the T group for mental adaptation and skills balance control movement were very high (5.37 and 3.30, respectively) and negligible in the C group (0.27 and 0.03, respectively). After 33 weeks of training, the swimming abilities of the participants from T were varied; four of them could swim the four styles, three could swim using the two styles, three could swim using one style, and three participants could swim at least 100 m without a rest, but their swimming technique was not good.

## 4. Discussion

The results of our study indicate that a 33-week swimming program is highly effective among adolescents with DS. First of all, despite of growing, the participants from the T group reduced their body mass, BMI, and body fat significantly while in the C group those indicators increased significantly. It is known that children and adolescents with DS have higher levels of total and regional fat mass than their non-DS peers [26,27,28]. Therefore, due to the higher percentage of body fat, there is a greater risk of many serious illnesses (e.g., coronary heart disease, hypertension, type 2 diabetes) [26,29]. Hence, it is very important to counteract obesity in the adolescents with DS and swimming therapy is a good, effective way of preventing and treating obesity. Our results are consistent with Suarez-Villadat et al. [30] conclusions, who showed that a 36-week swimming program resulted in a decrease in the levels of body fat and BMI in a sample of adolescents with DS. Moreover, Farías-Valenzuela et al. studies showed that 10 months activity program based on motor games can also reduce body fat in young adults with DS [31]. However, other studies have shown that a 21-week plyometric program did not lead to any changes in BMI and body fat of the participants with DS [32].

Relative VO_2_max is an important indicator of health status and is strongly associated with health and disease in adulthood [33]. Due to the autonomic dysfunction, reduced maximal heart rate, reduced ventilatory capacity, and metabolic dysfunction, children and adolescents with DS have lower cardiorespiratory fitness levels compared to their peers without DS, which can negatively interfere with their health and quality of life [10,34]. Therefore, to improve health and functionality of people with DS, aerobic training should be conducted. The results of our study indicate that 33 weeks swimming program significantly improved the aerobic capacity in the T group. During the same time, the aerobic capacity decreased in C. VO_2_max increased in the T group both in absolute (L/min) and relative values (mL/kg/min). In the C group the absolute values of VO_2_max did not change significantly, still the increase of body mass in this group caused a drop of VO_2_max expressed in relative values. Our results are consistent with Savucu [35] and Lewis et al. [36] who demonstrated significant improvement in VO_2_max following aerobic training in adolescent with DS. Moreover, Silva et al. [37] studies showed that Wii-based exercises also can improve aerobic capacity in adults with DS. Positive effects on cardiovascular fitness was also noted in children and adolescents with DS following 12-week Bharatnatyam-based dance therapy [38]. Other studies, however, did not confirm the positive impact of aerobic training on VO_2_max [14,17,39]. We would recommend applying a large, progressively increasing training volume to improve aerobic capacity in people with DS. Moreover, the training program must be attractive for participants, they cannot be bored during training. We believe that aerobic swimming training should be an integral part of the daily routine for people with DS.

Our study indicates that 33 weeks of swimming program can improve static arm strength, trunk strength, and endurance/functional strength in young people with DS. There were no significant changes in C. Swimming activates both abdominal and shoulder muscles, therefore changes in trunk strength and endurance/functional strength were expected following the intervention. However, an increase in handgrip strength noted in T was surprising. It is difficult to explain as in the course of the swimming program we did not apply exercises improving static arm strength. It is possible that increasing lean body mass might have influenced static arm strength in the T group, similarly to the participants tested by Çiğdem et al. [40]. We did not note improvements in arms speed movements, balance, and flexibility following rehabilitation. In contrast, Ilinca et al. [16] observed improvement in flexibility of young adults with DS following 24 weeks rehabilitation, the intervention, however, included stretching and balance exercises performed outside the pool.

The results of WOTA2 testing showed huge development of aquatic skills in adolescent with DS, both mental adaptation and skills balance control movement improved significantly in the T group. It is consisted with Vaščáková and Kudláček results who showed significant improvement of aquatic skills in children with different severe disabilities following 10 weeks Halliwick swimming intervention [41]. It should be noted that during the last 10 weeks of rehabilitation, the adolescents usually swam 700–1000 m. At the start of the program only four adolescents from the T group could swim using the improper technique (backstroke only). Following the intervention, all of them could swim backstroke, eight of them—freestyle, and three of them—breaststroke. What is very important for all participants from the training group, swimming program was very well tolerated. The participants enjoyed the swimming sessions. Also, the parents/guardians of the participants from the T group reported that their children were very satisfied to be able to participate in the project. Similar observations were made by Declerck et al. [42] who investigated enjoyment and specific benefits of a swimming program for youth with cerebral palsy.

## 5. Limitations and Strengths of the Study

The limitations of the study were: large age difference between participants; from 11 to 19 years old, small number of participants—11 in each group, and lack of intensity measurement of exercise during our swimming program. The strong points of the study were: adolescents participated regularly in the program with attendance above 90%, the participants were assessed with various tests (fitness assessment, aerobic fitness assessment, body composition assessment). Therefore, it was possible to evaluate the influence of the swimming program on the functionality of adolescents in various aspects.

## 6. Conclusions

Thirty-three weeks of swimming program resulted in the decreases in body mass, body fat, and BMI. During the experiment, the increases in the above-mentioned parameters were noted in the C group. Moreover, significant improvements in aerobic capacity and muscle strength were noted in T group. Aquatic skills also improved considerably in the training group. Changes in C were not significant. We strongly recommend the use of our swimming program as a highly effective and well tolerated form of exercises for adolescents with DS. It can be a good method to prevent diseases of civilization and specific diseases in youth with DS.

## Figures and Tables

**Table 1:** Mean and standard deviations for absolute values of body composition and aerobic capacity.

Group/Parameter		Height (m)	Body Mass (kg)	BMI	Fat (%)	Fat (kg)	VO_2_max (L/min)	VO_2_max (mL/kg/min)	HRmax
T	Before	1.50 ± 0.09	56.8 ± 7.97	25.1 ± 2.37	26.1± 4.23	15.1 ± 4.47	1.56 ± 0.33	27.4 ± 3.86	167 ± 9.66
	After	1.52 ± 0.08 *	55.0 ± 7.11 *	24.0 ± 2.05 *	23.7 ± 4.19 *	13.2 ± 3.92 *	1.74 ± 0.34 *	31.7 ± 4.05 *	168 ± 8.25
C	Before	1.50 ± 0.12 *	57.3 ± 8.43	25.4 ± 2.46	25.4 ± 3.32	14.5 ± 2.76	1.57 ± 0.35	27.2 ± 3.13	169 ± 5.79
	After	1.52 ± 0.11	59.7 ± 8.29 *	26.0 ± 2.72 *	26.8 ± 3.47 *	16.0 ± 3.11 *	1.55 ± 0.33	25.9 ± 3.08 *	169 ± 5.37

Notes: * significant difference from baseline, (*p* ≤ 0.05). BMI: body mass index VO_2_max: maximal oxygen uptake; HRmax: heart rate; T: training group; C: control group.

**Table 2:** Mean percentage changes, standard deviations, 95% confidence intervals, and effect sizes for body composition and aerobic capacity.

Group/Parameter		Height (m)	Body Mass (kg)	BMI	Fat (%)	Fat (kg)	VO_2_max (L/min)	VO_2_max (mL/kg/min)	HRmax (b/min)
T	% change	0.84 ± 0.61	−3.03± 1.60 *	−4.63 ± 1.80 *	−9.21 ± 5.89 *	−11.9 ± 6.64 *	12.9 ± 12.0 *	16.3 ± 11.6 *	0.16 ± 1.40
	95% CI	0.48 to 1.20	−3.98 to −2.08	−5.69 to −3.57	−12.7 to −5.73	−15.8 to −7.99	5.75 to 20.0	9.44 to 23.2	−0.67 to 0.99
T vs. C	ES	0.14	3.14	3.33	3.10	4.09	1.25	2.17	0.06
C	% change	0.98 ± 1.18	4.39 ± 2.79	2.38 ± 2.23	5.57 ± 2.70	10.2 ± 3.13	−0.54 ± 8.45	−4.80 ± 6.38	0.07 ± 1.55
	95% CI	0.28 to 1.68	2.74 to 6.04	1.06 to 3.70	3.97 to 7.17	8.35 to 12.0	−5.50 to 4.42	−8.57 to −1.03	−0.85 to 0.99

Note: * significant difference from the control, (*p* ≤ 0.05). ES: effect size.

**Table 3:** Mean and standard deviations for absolute values of Eurofit parameters.

Group/Muscle		Speed of Limb (reps)	Handgrip (kg)	Balance (No of Contacts)	Flexibility (cm)	Sit-Ups (reps)	Arms Strength-Endurance (s)
T	Before	26.7± 9.91	17.1 ± 5.42	13.7 ± 2.00	11.0 ± 9.09	13.4 ± 2.90	2.79 ± 2.89
	After	26.6 ± 9.18	22.3 ± 5.63 *	10.6 ± 8.39	12.9 ± 8.39	16.3 ± 2.89 *	5.77 ± 2.57 *
C	Before	29.0 ± 8.08	16.5 ± 3.54	14.8 ± 2.95	10.9 ± 6.12	14.0 ± 2.63	3.20 ± 3.06
	After	28.8 ± 8.49	16.8 ± 4.41	14.0 ± 3.28	11.7 ± 8.51	14.2 ± 2.62	3.60 ± 2.40

Note: * significant difference from baseline, (*p* ≤ 0.05).

## Data Availability

The data presented in this study are available on request from the corresponding author. The data are not publicly available due to its processing by the founder.

## References

[1] Bertapelli F., Pitetti K., Agiovlasitis S., Guerra-Junior G. (2016). Overweight and obesity in children and adolescents with Down syndrome-prevalence, determinants, consequences, and interventions: A literature review. Res. Dev. Disabil..

[2] World Health Organization (2009). Global Health Risks Mortality and Burden of Disease Attributable to Selected Major Risks.

[3] Vis J.C., Duffels M.G.J., Winter M.M., Weijerman M.E., Cobben J.M., Huisman S.A., Mulder B.J.M. (2009). Down syndrome: A cardiovascular perspective. J. Intellect. Disabil. Res..

[4] Heller T., Hsieh K., Rimmer J. (2008). Barriers and support for exercise participation among adults with Down syndrome. J. Gerontol. Soc. Work.

[5] Castro-Piñero J., Carbonell-Baeza A., Martinez-Gomez D., Gómez-Martínez S., Cabanas-Sánchez V., Santiago C., Veses A.N., Bandrés F., Gonzalez-Galo A., Gomez-Gallego F. (2014). Follow-up in healthyschoolchildren and in adolescents with DOWN syndrome: Psycho-environmental and genetic determinants of physical activity andits impact on fitness, cardiovascular diseases, inflammatory biomarkersand mental health; the UP&DOWN Study. BMC Public Health.

[6] Fernhall B., McCubbin J.A., Pitetti K.H., Rintala P., Rimmer J.H., Millar A.L., De Silva A. (2001). Prediction of maximal heart rate in individuals with mental retardation. J. Sci. Med. Sports.

[7] Wee S.O., Pitetti K.H., Goulopoulou S., Collier S.R., Guerra M., Baynard M. (2015). Impact of obesity and Down syndrome on peak heart rate and aerobic capacity in youth and adults. Res. Dev. Disabil..

[8] Hayden M.F. (1998). Mortality among people with mental retardation living in the united states: Research review and policy application. Ment. Retard..

[9] Izquierdo-Gomez R., Martinez-Gomez D., Villagra A., Fernhall B., Veiga O.L. (2015). Associations of physical activity with fatness and fitness in adolescents with Down syndrome: The UP & DOWN study. Res. Dev. Disabil..

[10] Pitetti K., Baynard T., Agiovlasitis S. (2013). Children and adolescents with Down syndrome, physical fitness and physical activity. J. Sports Health. Sci..

[11] DHSC (2019). UK Chief Medical Officers Physical Activity Guidelines.

[12] Guthold R., Stevens G.A., Riley L.M., Bull F.C. (2020). Global Trends in Insufficient Physical Activity among Adolescents: A Pooled Analysis of 298 Population-Based Surveys with 16 Million Participants. Lancet Child Adolesc. Health.

[13] Shields N., Plant S., Warren C., Wollersheim D., Peiris C. (2018). Do adults with Down syndrome do the same amount of physical activity as adults without disability? A proof of principle study. J. Appl. Res. Intellect. Disabil..

[14] Seron B.B., Modesto E.L., Stanganelli L.C.R., Oliveira de Carvalho E.M., Greguol M. (2017). Effects of aerobic and resistance training on the cardiorespiratory fitness of young people with Down Syndrome. Rev. Bras. Cineantropom. Hum..

[15] Paul Y., Ellapen T.J., Barnard M., Hammill H.V., Swanepoel M. (2019). The health benefits of exercise therapy for patients with Down syndrome: A systematic review. Afr. J. Disabil..

[16] Ilinca I., Roșulescu E., Cosma G., Danoiu M. (2019). The influence of physiotherapy on functional fitness of adults with down syndrome. Phys. Ed. Sport Kin. J..

[17] Millar A.L., Fernhall B., Burkett L.N. (1993). Effects of aerobic training in adolescents with Down syndrome. Med. Sci. Sports Exerc..

[18] Pérez C.A., Carral J.M.C., Costas A.A., Martínez S.V., Martínez-Lemos R.I. (2018). Water-based exercise for adults with Down syndrome: Findings from a preliminary study. Int. J. Ther. Rehabil..

[19] Suarez-Villadat B., Veiga O.L., Villagraa A.Z., Izquierdo-Gomez R.P., UP&DOWN Study Group (2019). Changes in Body Composition and Physical Fitness in Adolescents with Down Syndrome: The UP&DOWN Longitudinal Study. Child. Obes..

[20] MacDonncha C., Watson A.S., McSweeney T., O’Donovan D. (1999). Reliability of Eurofit physical items for adolescent males with and without mental retardation. Adapt. Phys. Act. Q..

[21] Baynard T., Pitetti K.H., Guerra M., Unnithan V.B., Fernhall B. (2008). Age-Related Changes in Aerobic Capacityin Individuals with Mental Retardation: A 20-yr Review. Med. Sci. Sports Exerc..

[22] Fernhall B., Pitetti K.H., Rimmer J.H., McCubbin J.A., Rintala P., Millar A.L., Kittredge J., Burkett L.N. (1996). Cardiorespiratory capacity of individuals with mental retardation including Down syndrome. Med. Sci. Sports Exerc..

[23] The American Academy for Cerebral Palsy and Developmental Medicine. https://www.aacpdm.org/UserFiles/file/BRK9c_Tirosh.pdf.

[24] Tirosh R., Katz-Leurer M., Getz M.D. (2008). Halliwick-Based Aquatic Assessments: Reliability and Validity. Int. J. Aquat. Res. Educ..

[25] Cohen J. (1988). Statistical Power Analysis for the Behavioral Sciences.

[26] González-Agüero A., Ara I., Moreno L.A., Vicente-Rodríguez G., Casajús J.A. (2011). Fat and lean masses in youths with Down syndrome: Gender differences. Res. Dev. Disabil..

[27] Murray J., Ryan-Krause P. (2010). Obesity in children with down syndrome: Background and recommendations for management. Pediatr. Nurs..

[28] O’Shea M., O’Shea C., Gibson L., Leo J., Carty C. (2018). The prevalence of obesity in children and young people with Down syndrome. J. Appl. Res. Intellect. Disabil..

[29] Maffeis C., Tato L. (2001). Long-term effects of childhood obesity on morbidity and mortality. Horm. Res..

[30] Suarez-Villadat B., Luna-Olivac L., Acebesa C., Villagraa A. (2020). The effect of swimming program on body composition levels in adolescents with Down syndrome. Res. Dev. Disabil..

[31] Farías-Valenzuela C., Cofré-Bolados C., Ferrari G., Espoz-Lazo S., Arenas-Sánchez G., Álvarez-Arangua S., Espinoza-Salinas A., Valdivia-Moral P. (2021). Effects of Motor-Games-Based Concurrent Training Program on Body Composition Indicators of Chilean Adults with Down Syndrome. Sustainability.

[32] González-Agüero A., Vicente-Rodríguez G., Gómez-Cabello A., Ara I., Moreno L.A., Casajús J.A. (2011). A combined training intervention programme increases lean mass in youths with Down syndrome. Res. Dev. Disabil..

[33] Paffenbarger R.S., Blair S.N., Lee I.M. (2001). A history of physical activity, cardiovascular health and longevity: The scientific cbcontributions of Jeremy N Morris, DSc, DPH, FRCP. Int. J. Epidemiol..

[34] González-Agüero A., Vicente-Rodriguez G., Moreno L.A., Guerra-Balic M., Ara I., Casajus J.A. (2010). Health-related physical fitness in children and adolescents with Down syndrome and response to training. Scand. J. Med. Sci. Sports.

[35] Savucu Y. (2010). Influence of 12-Week Training on Aerobic Capacity and Respiratory Functions of Adolescents with down Syndrome. World Appl. Sci. J..

[36] Lewis C.L., Fragala-Pinkham M.A. (2005). Effects of aerobic conditioning and strength training on a child with Down syndrome: A case study. Pediatr. Phys. Ther..

[37] Silva V., Campos C., Sá A., Cavadas M., Pinto J., Simões P., Machado S., Murillo-Rodríguez E., Barbosa-Rocha N. (2017). Wii-based exercise program to improve physical fitness, motor proficiency and functional mobility in adults with Down syndrome. J. Intellectx. Disabil. Res..

[38] Parab S., Bose M., Shayer S., Saini R.K., Salvi M., Ravi P., Sawant P. (2019). Bharatnatyam-based dance therapy in Children and and Adolescents with Down Syndrome. Clin. Kinesiol..

[39] Varela A.M., Sardinha L.B., Pitetti K.H. (2001). Effects of an aerobic rowing training regimen in young adults with Down syndrome. Am. J. Ment. Retard..

[40] Çiğdem E., Hüzmeli E.D., Gökçek Ö. (2020). Investigation of the Relationship between Physical Activity and Body Mass Index in Children with Down Syndrome. J. Pediatr. Res..

[41] Vaščáková T., Kudláček M. (2015). Halliwick Concept of Swimming and its Influence on Motoric Competencies of Children with Severe Disabilities. Eur. J. Adapt. Phys. Act..

[42] Declerck M., Verheul M., Daly D., Sanders R. (2016). Benefits and Enjoyment of a Swimming Intervention for Youth with Cerebral Palsy: An RCT Study. Pediatr. Phys. Ther..

